# Rabies in the Republic of Kazakhstan: spatial and temporal characteristics of disease spread over one decade (2013–2022)

**DOI:** 10.3389/fvets.2023.1252265

**Published:** 2023-09-05

**Authors:** Anar M. Kabzhanova, Ablaikhan S. Kadyrov, Aizada A. Mukhanbetkaliyeva, Gulzhan N. Yessembekova, Yersin Y. Mukhanbetkaliyev, Fedor I. Korennoy, Andres M. Perez, Sarsenbay K. Abdrakhmanov

**Affiliations:** ^1^Faculty of Veterinary and Livestock Technology, S. Seifullin Kazakh Agro Technical Research University, Astana, Kazakhstan; ^2^Federal Centre for Animal Health (FGBI ARRIAH), Vladimir, Russia; ^3^Center for Animal Health and Food Safety, College of Veterinary Medicine, University of Minnesota, St. Paul Campus, St. Paul, MN, United States

**Keywords:** spatial clustering, rabies, Kazakhstan, epidemiology, disease control, spatial scan statistic

## Abstract

Rabies is a fatal zoonotic disease that remains endemic in Kazakhstan despite the implementation of annual vaccination campaigns. Using data collected over a 10-year time period, the objective of this study was to provide updated information on the epidemiological situation of the disease in the country, and quantitative data on the species-specific spatial distribution of rabies and on the epidemiological features associated with that clustering. Five significant (*p* < 0.05) clusters of disease were detected. Clusters in southern Kazakhstan were associated with companion animals, which are likely explained by the maintenance of a domestic cycle of the disease in the most densely populated region of the country. Livestock cases were most frequent in clusters in the eastern (where wildlife cases were also frequent) and western regions of Kazakhstan, with higher probability of occurrence in spring and summer, compared to the rest of the year. The results here are consistent with differential patterns for disease transmission in Kazakhstan and will contribute to the design and implementation of zoning approaches to support the progressive control of rabies in the country.

## 1. Introduction

Rabies is a viral disease that affects the central nervous system of mammals, including humans. The rabies virus is particularly present in the saliva and brain of infected animals, most commonly dogs, and it is transmitted by bites. Bats also represent an important reservoir in certain regions (1). Prevalence of the disease in wildlife species creates multiple opportunities for cross-species transmission, mostly affecting domestic animals and humans. The rabies virus belongs to the Mononegavirales order, which are viruses with a nonsegmented, negative-stranded RNA genome. Within this group, viruses with a distinct “bullet” shape are grouped into the Rhabdoviridae family, which are subsequently divided in at least three genera of animal viruses referred to as Lyssavirus (that includes the rabies virus), Ephemerovirus, and Vesiculovirus. The rabies virus is transmitted through the direct contact of broken skin or mucous membranes (eyes, nose, or mouth) with saliva or nervous system tissue from an infected animal (2).

Rabies is believed to be present in >150 countries, with most cases reported in rural, underdeveloped areas of Africa and Asia (3). The disease severely affects the public health and social development of infected regions. The increase observed in of dog rabies cases across much of Asia and Africa has been attributed to the low priority given to control of the disease, associated with lack of awareness of the true scale and magnitude of the disease burden as well as misperceptions as to the feasibility, cost-effectiveness, and public health benefits of dog rabies control (4) Rabies also results in indirect costs for the veterinary and medical sectors associated with disease monitoring and implementation of preventive measures and treatments. The disease also impacts livestock production, negatively affecting the country’s economy and business entities (5–8).

Rabies is a serious public health concern in Kazakhstan (9, 10). The cumulative mortality rate, considering the number of human deaths recorded between 2010 and 2017 and the population size registered in 2020, attributable to rabies was believed to be higher in Kazakhstan (*n* = 38; 2 per million inhabitants) than in neighboring countries, such as Uzbekistan (no death recorded), Kyrgyzstan (*n* = 12; 1.8 per million), Turkmenistan (*n* = 1; 0.2 per million), and the Russian Federation (*n* = 51; 0.3 per million), with the only exception of China (*n* = 9,197 deaths, 6.6 per million) (11). Although, human deaths were not recorded in Kazakhstan between 2018 and 2021, unfortunately (11). Most (725/1,119) animal cases reported between 1996 and 2004 in Kazakhstan affected cattle, causing far-reaching economic losses (12, 13). However, because of inter-species transmission, prevention measures targeting only domestic animals are insufficient to eradicate rabies and protect the human population, leaving different parts of the country vulnerable to the disease (14–16). Key measures taken to control the disease in Kazakhstan include (1) strategic oral vaccination of wild animals during outbreaks and in areas of potential disease spread; (2) compulsory and prophylactic vaccination of prone domestic animals; (3) registration of susceptible domestic animals, and (4) public education and outreach (16, 17). The specifications of the vaccination campaign, including the number of vaccine doses and vaccine type used, depend on the total number of susceptible livestock and the epidemiological situation of the district (16–19). Briefly, all susceptible livestock, dogs, and cats are vaccinated, and the oral vaccine is distributed for wild animals, in high-risk districts, whereas vaccination and surveillance activities are selectively applied in districts classified as mid, low, or negligible risk (18–21).

Despite efforts to control the disease, rabies is still endemic in Kazakhstan. Reasons that may explain the limited success in controlling the disease in the country include, for example, challenges associated with the identification of the location and size of the susceptible population (which may result in incomplete vaccination coverage), limitations on the implementation of control measures (given that the coordination of public service actions to diagnose and eliminate the outbreaks may take up to 7 days), the influence of detection and information bias, and limited epidemiological surveillance, particularly in wild animals (10, 19, 20).

Wildlife species or dogs can serve as reservoirs for rabies and spill over into livestock and humans. This results in two epidemiological cycles for disease transmission, referred to as wildlife, sylvatic, or rural, and domestic animal or urban, respectively. Both cycles overlap, sometimes, in locations and periods of time when wild life species and dogs co-exist. Under certain conditions, they may be exacerbated by changes of human activities and climate change (22).

Kazakhstan is the ninth largest (by area) country in the world. On its vast and diverse geography, rabies cases are historically reported in a variety of domestic and wild life species (23, 24). A retrospective analysis of data provided by the National Veterinary Services, resulted in the identification of significant spatial and temporal clusters, and zoning and regionalization strategies were subsequently recommended (19). Although the frequency of cases per susceptible species in Kazakhstan seems to also vary across regions (10, 17, 19), species-specific clusters of disease, and their relation with demographic and environmental factors, are yet to be elucidated.

The objectives of this study were to provide updated information on the epidemiological situation of the disease in the country. Additionally, it aimed to identify spatial clusters of rabies cases stratified per group of susceptible species, and to characterize those clusters. Researchers’ findings will help inform decisions related to disease prevention and control in Kazakhstan and other Central Asia countries within a regional initiative, which is being implemented by the members of an informal network MEEREB (Middle East, Eastern Europe and Central Asia) countries (25).

## 2. Materials and methods

A database including all confirmed animal rabies cases reported in Kazakhstan between 2013 and 2022 was obtained from the Committee for Veterinary Control and Supervision of the Ministry of Agriculture of the Republic of Kazakhstan. For each reported case, the database included information on the date of reporting, location (latitude, longitude), and species affected. Rabies cases were grouped into one of three possible categories, depending on the affected species, as livestock (cattle, horses, sheep, camels, mules), companion (dogs and cats), or wild life (foxes, wolves, jackals).

Spatial clusters of cases were identified using the multinomial model of the spatial scan statistic, implemented in the SatScan software (26). Briefly, circular windows were alternatively imposed over all locations in which rabies cases were reported, with the radius of the window varying up to a maximum of 50% of the cases. Distances are computed over the surface of the Earth and for that reason, there is no need to project the data. Each window represents a candidate cluster, in which the number of cases observed for each category (livestock, companion, wildlife) was compared with the expected under the null hypothesis of even distribution of cases per category in space. The significance of each candidate cluster was evaluated using a likelihood ratio test estimated after successive Monte Carlo simulations (*n* = 999).

Each significant (*p* < 0.01) cluster was characterized and described by:

- the identification of the group of susceptible species for which the number of observed cases in the cluster was higher than the expected under the null hypothesis of even distribution of cases (O/E),
- the estimation of the percentage of cases for each month accumulated through the duration of the cluster; additionally, for clusters in which the value of O/E was higher than 1 for more than one group of species, the cross-correlation in the number of cases reported between those species at different time lags was also computed, and
- the estimation of the human population density (persons/km^2^) within the cluster, which was computed by averaging the zonal statistics using the Gridded Population of the World raster data for 2020 with 30 arc-second spatial resolution.^1^

## 3. Results

The total number of outbreaks, defined as one or more rabies cases identified from any given location (coordinate) at any given day, reported over the 10 year period assessed here was 1,016, including 1,334 animal cases. The largest number of outbreaks was recorded in 2013 (*n* = 140), 2015 (*n* = 140), and 2020 (*n* = 124). Most cases (*n* = 889, 66.6%) were reported in livestock, whereas 395 (29.6%) cases affected companion animals, and the remaining 50 (3.7%) cases were associated with wildlife species.

**Figure 1:**
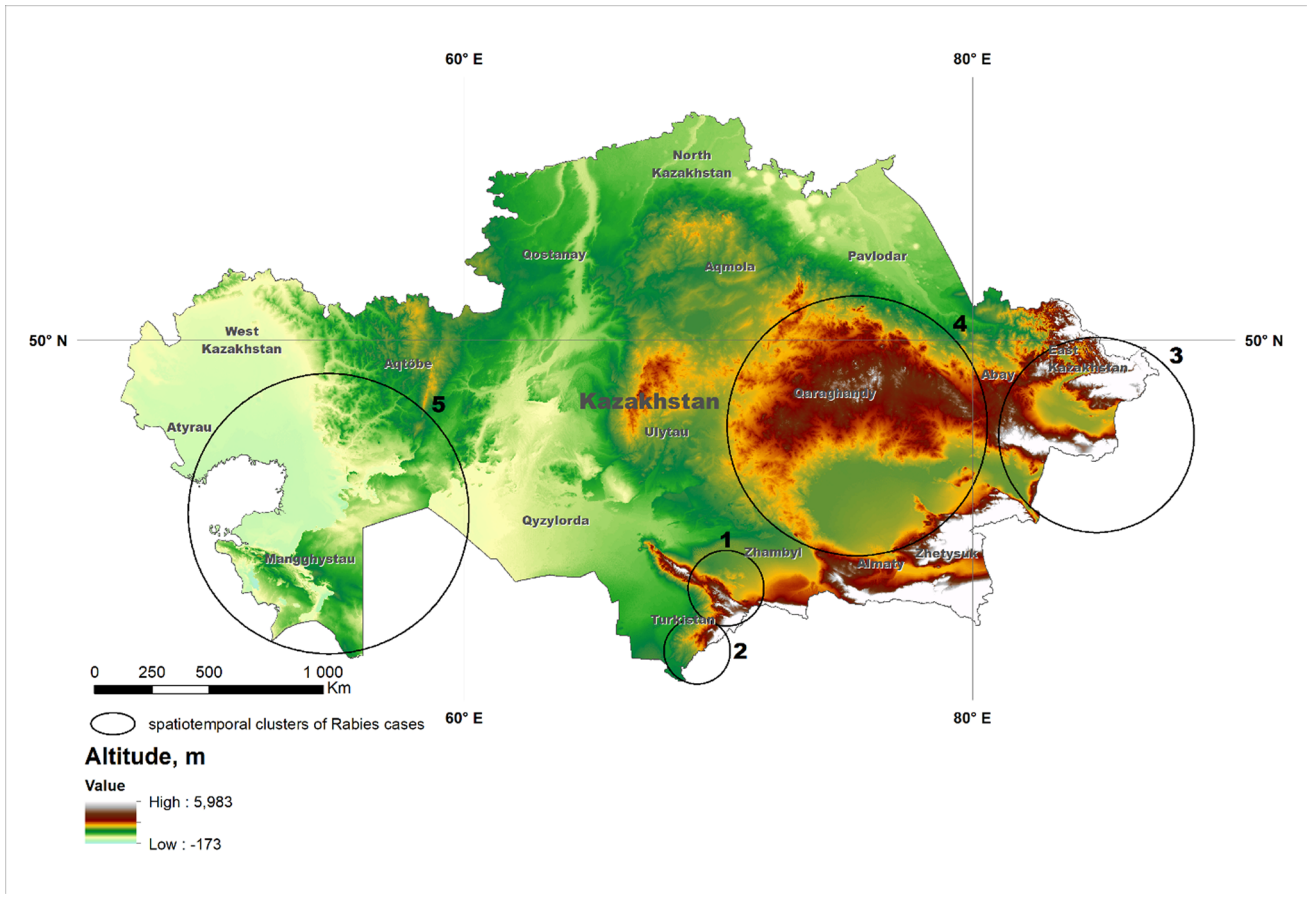
Significant (*p* < 0.01) clusters of rabies cases reported in Kazakhstan from January 2013 through December 2022 identified using the multinomial model of the spatial scan statistic. Details of the clusters are provided in Table 1. The background shade indicates the altitude of the location.

The use of the multinomial scan test resulted in the identification of 5 spatial clusters (Figure 1). In four of the five clusters, values of O/E > 1 were estimated for one single group of species (two for companion animals, two for livestock), whereas values of O/E > 1 in two group of species (livestock and wildlife) were estimated in the remaining cluster (Table 1).

**Table 1:** Significant (*p* < 0.01) clusters of rabies cases reported in Kazakhstan from January 2013 through December 2022 adjusted by type of susceptible species (categorized as livestock, companion animals, and wildlife) identified using the multinomial model of the spatial scan statistic.

ID	No. of cases	Latitude	Longitude	Radius (km)	Observed-to-expected ratio [number of observed cases]		
					Livestock	Companion	Wildlife
1	83	43.289760	70.306537	120.7	0.24 [11]	**2.18 [70]**	0.47 [2]
2	87	41.455378	69.166534	108.7	0.31 [15]	**2.11 [71]**	0.22 [1]
3	67	47.479786	84.877645	288.6	**1.67 [63]**	0.08 [2]	0.56 [2]
4	64	47.666540	75.466459	382.9	**1.61 [58]**	0.20 [5]	0.30 [1]
5	88	45.267864	54.676539	431.5	**1.36 [67]**	0.25 [12]	**1.98 [9]**

Observed-to-expected ratios higher than 1 are bolded.

**Figure 2:**
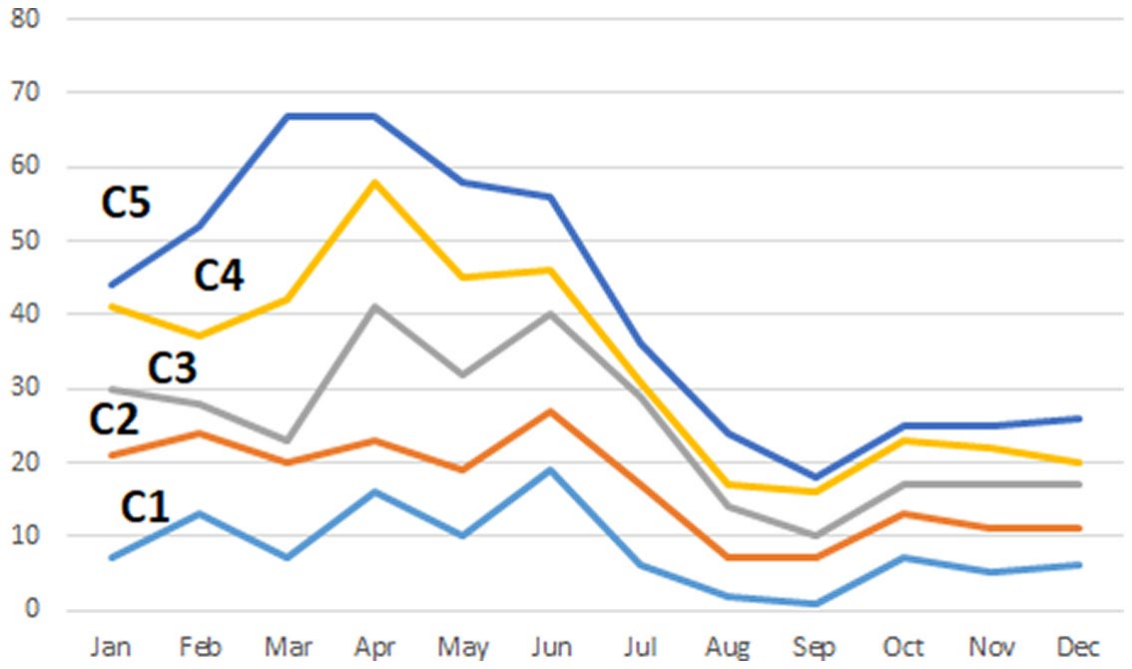
Percentage of rabies cases reported per month on each of five significant (*p* < 0.01) clusters (C1–C5) detected in Kazakhstan from January 2013 through December 2022 identified using the multinomial model of the spatial scan statistic. Details of the clusters are provided in Table 1.

**Figure 3:**
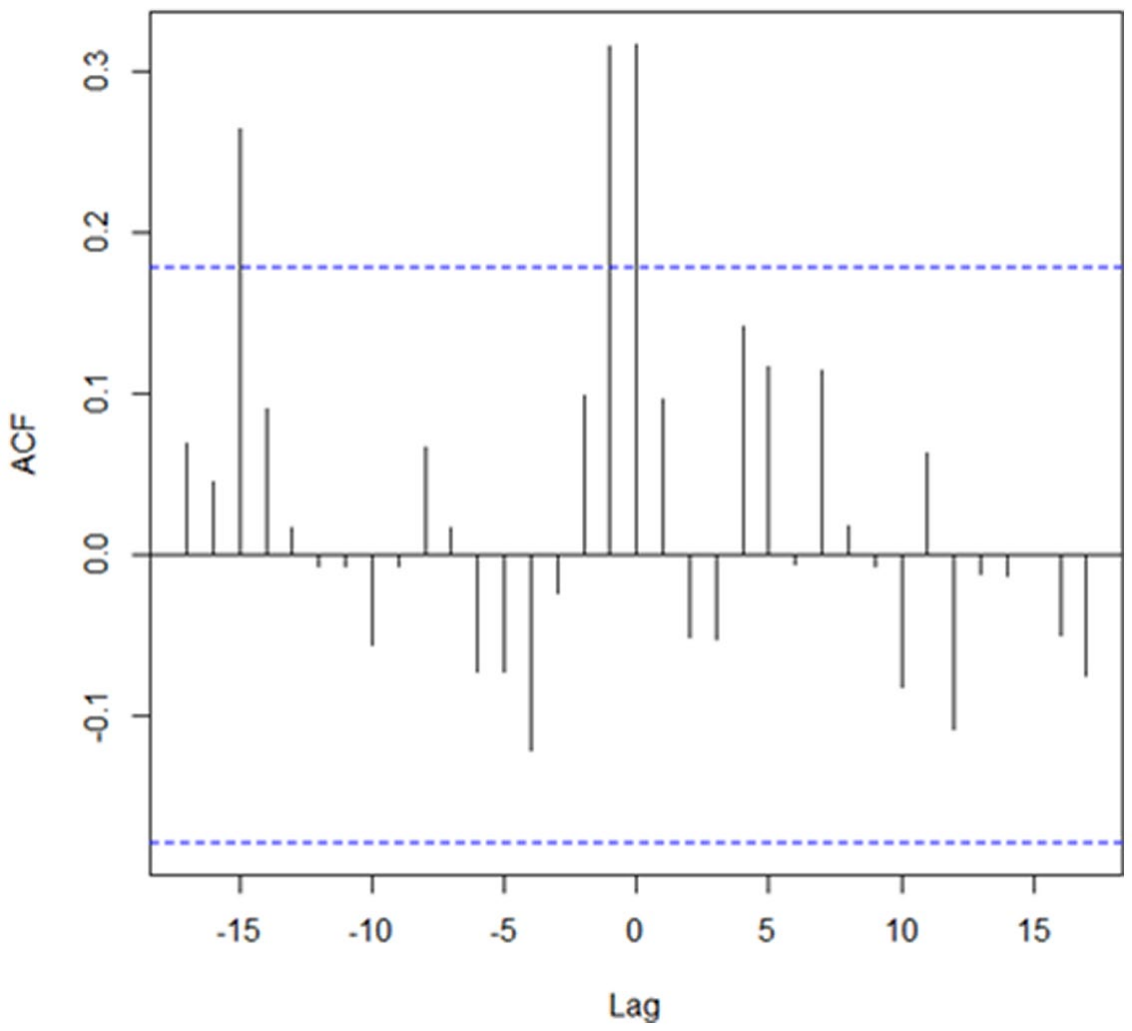
Cross-autocorrelation (ACF) between the number of cases reported in livestock and wildlife in the only significant (*p* < 0.01) cluster detected in Kazakhstan from January 2013 through December 2022 in which there were more observed than the expected in two groups of species. Dashed lines denote 95% confidence interval boundaries. Details of this cluster, denoted as cluster #5, are provided in Table 1.

The highest human population density was estimated in clusters #1 and #2 (35.6 and 64.0 persons/km^2^, respectively), whereas it was substantially lower in clusters #3, #4, and #5 (3.1, 4.0, and 3.6 persons/km^2^, respectively). The proportion of cumulative cases on each clusters was highest during Spring and Summer month, with the highest seasonal difference observed in clusters in which values of O/E > 1 were found for agricultural species (clusters #3, #4, and #5), whereas the difference was not so pronounced for clusters in which the O/E value was highest for companion animals (clusters #1 and #2; Figure 2). The cross-correlation for cases in wildlife and livestock was computed for the only cluster in which more than one group of species resulted in values of O/E > 1 with significant associations estimated at lags of 0, −1, and −15 (Figure 3).

## 4. Discussion

Rabies is a fatal zoonotic disease that is endemic to Kazakhstan. The analysis of the evolution of the number of cases over time and their spatial distribution revealed an heterogeneous pattern likely associated with environmental conditions and demographic factors (5, 6, 11, 14). The results presented here provide updated information on the situation of the disease in the country, contribute with quantitative measures of the species-specific clusters of disease, and on the features associated with the clustering.

The distinct temporal and spatial patterns of species-specific clusters reported here are likely associated with disparate cycles of rabies transmission in Kazakhstan.

Clusters in the southern part of the country (denoted as #1 and #2; Figure 1) were associated with companion animals, as indicated by the value of the observed-to-expected ratio (Table 1). Although cases in those clusters were more frequent in spring and summer than in winter and fall, probably due to a higher probability of inter-species interaction when weather is mild compared to extreme conditions, that difference was not as pronounced as in other disease clusters (Figure 2). Because of the relatively mild weather conditions, high human population density, and the subsequent availability of food and shelter, stray dogs are relatively frequent in this part of the country. These findings suggest that the dynamics of rabies transmission in southern Kazakhstan mostly follows an urban cycle, associated with a largest human population density and cases observed only in companion animals. Free-ranging companion dogs, also referred to as “stray dogs,” impose a risk for disease spread in this part of the country, because of the risk for contact with wildlife species (27, 28). Identification of the distribution and location of the susceptible canine population, sterilization and castration, evaluation of aggressive dogs, outreach and education of the public, and systematic vaccination are recommended activities in these conditions (28–31).

Clusters in eastern Kazakhstan (#3, #4), in turn, were mostly associated with livestock, and specifically, with cattle (86 and 88% of the observed cases on each cluster, respectively). The region is characterized by low human population density and a rural landscape that is home to the largest population of livestock in the country. The region covered by cluster #4 (Figure 1) is characterized by cold winters and deep snow cover and for that reason, livestock is typically kept within farms in winter. In association with that practice, most rabies cases occurred in spring and summer, when animals are grazed on pastures in which contact with infected animals is likely to occur. Most notably, only few wild life cases were reported in these clusters and it is possible that the landscape, characterized by a steep terrain, may impair the ability to detect and report cases in wild life. In turn, the region in which cluster #3 was located is represented by vast steppe territories suitable for animal husbandry. Much of this territory is characterized by relatively mild winters and low precipitation, keeping farm animals on pastures (Zhailau) far from settlements, and associated with transhumance practices. The population density is also low, with the exception of suburbs and regional centers. In this environment, a heavy involvement of the veterinary services to early detect high risk contacts between wildlife and livestock is mostly important (29–31).

Finally, cluster #5 in Western Kazakhstan was the only one in which values of O/E > 1 was estimated in more than one group animal species, namely, livestock and wildlife. Cases in wild life tend to occur at the same time or 1 month earlier than cases in livestock (Figure 3) suggesting that there was an association in the transmission of the disease between groups of species, which is consistent with the definition of a sylvatic cycle. This was also the cluster with the largest number of cases in wild animals (*n* = 9), including 7 foxes and 2 wolves. Because of the landscape, wildlife cases may be easier to observe in this region compared to clusters 3 and 4. This is also the cluster with the largest monthly difference in the reporting of cases, with most cases occurring in spring and summer (Figure 2), which may be exacerbated by heat waves from the Caspian Sea that impact on this during those seasons. Despite the evidence of cases of rabies in farm animals associated with cases of wildlife, which are particularly sensitive to changes in natural and climatic parameters such as temperature, humidity, precipitation, the importance of these factors is often overlooked (5, 11).

The presence of a wildlife reservoir is probably the most important factor in common between the detected clusters. Wildlife species affected by rabies in Kazakhstan includes foxes, wolves, and jackals, and the opportunity for contacting with livestock and companion animals usually occurs in the cold season, in which wild life get closer to human settlements in a search for protection and food. This situation typically results in an increase of rabies cases reported in spring.

In summary, this paper presents updated information on the epidemiological dynamics of rabies in Kazakhstan, and also, provides quantitative evidence that supports the hypothesis of alternative transmission cycles (urban in the south, rural in the east and west) in the country. The results support the implementation of differential control and surveillance strategies for rabies in Kazakhstan and provide baseline information that will be helpful in measuring the success of prevention programs.

## Data availability statement

The data analyzed in this study is subject to the following licenses/restrictions: data are property of the government of Kazakhstan and may be available upon request. Requests to access these datasets should be directed to S. Seifullin Kazakh Agro Technical University, Astana; s_abdrakhmanov@mail.ru.

## Author contributions

SA, FK, and AP: conceptualization. AP: methodology. AnK and AbK: software. AM, GY, and YM: data curation. AnK: writing—original draft preparation. AP and SA: writing—review and editing. FK: visualization. SA: supervision, project administration, and funding acquisition. All authors contributed to the article and approved the submitted version.

## Funding

This research was funded by the Government of Kazakhstan, grant # AP19679670 “Improvement preventive measures against infectious diseases of animals (on the rabies example), based on using of information and communication technologies.”

## Conflict of interest

The authors declare that the research was conducted in the absence of any commercial or financial relationships that could be construed as a potential conflict of interest.

## Publisher’s note

All claims expressed in this article are solely those of the authors and do not necessarily represent those of their affiliated organizations, or those of the publisher, the editors and the reviewers. Any product that may be evaluated in this article, or claim that may be made by its manufacturer, is not guaranteed or endorsed by the publisher.

## Footnotes

^1^ https://sedac.ciesin.columbia.edu/data/collection/gpw-v3
